# The Anti-Melanogenic Effects of Ganodermanontriol from the Medicinal Mushroom *Ganoderma lucidum* through the Regulation of the CREB and MAPK Signaling Pathways in B16F10 Cells

**DOI:** 10.3390/molecules29163976

**Published:** 2024-08-22

**Authors:** Che-Hwon Park, Youn-Lee Oh, Ju-Hyeon Shin, Young-Jin Park

**Affiliations:** 1 Department of Medicinal Biosciences, Research Institute for Biomedical & Health Science, College of Biomedical and Health Science, Konkuk University, 268 Chungwon-daero, Chungju-si 27478, Republic of Korea; chehwon9798@kku.ac.kr (C.-H.P.); shin99@kku.ac.kr (J.-H.S.); 2 Mushroom Research Division, National Institute of Horticultural and Herbal Science, Rural Development Administration, 92, Bisan-ro, Eumseong-gun 27709, Republic of Korea

**Keywords:** B16F10 melanoma cells, *Ganoderma lucidum*, ganodermanontriol, microphthalmia-associated transcription factor, tyrosinase, tyrosinase-related proteins

## Abstract

*Ganoderma lucidum*, a member of the Basidiomycetes family, is attracting attention for its medicinal potential due to its biological activity and the presence of numerous bioactive compounds. Although it is known that extracts of this mushroom inhibit melanin production, there are few reports on a single substance associated with this effect. In this study, we identified ganodermanontriol (GT), a novel compound from *G. lucidum*, that effectively inhibited melanin biosynthesis in B16F10 cells. GT inhibits melanin production by suppressing the expression of cellular tyrosinase proteins and microphthalmia-related transcription factor (MITF). Furthermore, GT affects the phosphorylation of cyclic adenosine monophosphate (cAMP) response element-binding protein (CREB) and mitogen-activated protein kinase (MAPK) signaling molecules, which are involved in melanogenesis in B16F10 cells. Finally, the biosynthesis of GT and other substances by *G. lucidum* was evaluated using HPLC analysis. Thus, this study revealed the mechanism by which GT in *G. lucidum* inhibits melanin production in B16F10 cells, and these findings will contribute to promoting the potential use of this mushroom in the future.

## 1. Introduction

Melanin, a black pigment synthesized from tyrosine by melanocytes, plays a crucial role in determining hair and skin color [1,2,3,4,5,6]. Melanogenesis is orchestrated by the key enzyme, tyrosinase (EC 1.14.18.1), a multifunctional copper-containing oxidase. Tyrosinase catalyzes the initial steps of melanin production by converting tyrosine into DOPA and subsequently into DOPAquinone, which undergoes further transformation to form melanin pigments [7]. Melanocytes, located in the basal epidermis and hair follicles, are responsible for pigment production and serve as natural ultraviolet radiation filters, protecting the skin from ultraviolet (UV) damage [1,8]. The melanogenesis pathway involves other critical enzymes, including tyrosinase-related protein 1 (TRP1) and tyrosinase-related protein 2 (TRP2), which further modify melanin intermediates to produce different types of melanin [1]. The factors influencing melanin production include genetics, UV radiation (UVR), and inflammation, with the melanocortin 1 receptor (MC1R) and microphthalmia-associated transcription factor (MITF) playing central roles in regulating this process [9,10].

*Ganoderma lucidum* has been used medicinally for a long time in Korea, China, and Japan [11]. Previous studies have reported various biological activities of these mushrooms, including antitumor, anti-inflammatory, antimutagenic, and antioxidant activities, and they are attracting attention as medicinal substances [12,13]. Thus, *G. lucidum* can be utilized as a food additive and an active pharmaceutical ingredient and has great potential as a cosmetic ingredient. The name *Ganoderma* is derived from the Greek ganos (meaning “brightness” and “sheen”) and derma (meaning “skin”). It has previously been reported that extracts of *G. lucidum* inhibit tyrosinase activity [14]. For the pharmacological and pharmaceutical use of natural products, it is important to identify specific single substances. Previous studies have reported that ganodermanontriol (GT) is a typical constituent in *G. lucidum* and has an inhibitory effect on human immunodeficiency virus type 1 (HIV-1) [15,16,17,18]. In addition, we previously reported the inhibitory effect of ganodermanondiol (GN), a triterpenoid derived from *G. lucidum*, on melanin biosynthesis in B16F10 cells [19]. However, except for GN, there have been no reports of compounds derived from *G. lucidum* that inhibit melanin biosynthesis.

Thus, this study aimed to identify a novel substance from *G. lucidum* that inhibits melanin production in B16F10 cells. In the present study, we evaluated the effects of GT, a triterpenoid isolated from *G. lucidum*, on melanogenesis-related proteins and mitogen-activated protein kinase (MAPK) signaling molecules in B16F10 cells. GT significantly inhibited the expression of tyrosinase-related proteins and the expression of microphthalmia-related transcription factor (MITF) in the B16F10 cells. Additionally, we found that this compound affected the phosphorylation of cAMP response element-binding protein (CREB), as well as MAPK signaling molecules, including extracellular signal-regulated kinase (ERK), c-Jun N-terminal kinase (JNK), and p38.

## 2. Results

### 2.1. Ganodermanontriol Inhibited Melanin Synthesis of B16F10 Cells

**Figure 1:**
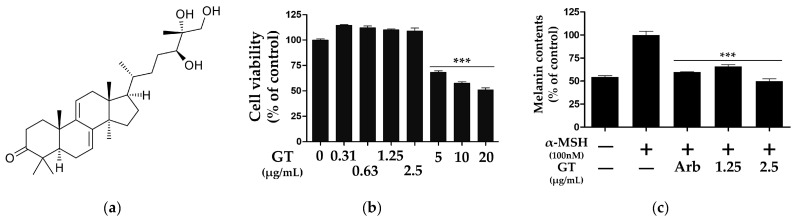
Chemical structure (**a**), cytotoxicity (**b**), and melanin synthesis inhibitory activity (**c**) of ganodermanontriol (GT). The data were analyzed using one-way ANOVA followed by Tukey’s test. *** *p* < 0.001 vs. B16F10 cells without sample treatment. Arb, arbutin (0.5 mM).

Ganodermanontriol (C_30_H_48_O_4_) has a triterpenoid structure and is one of the major active compounds in *G. lucidum* [15,16,17,18] (Figure 1a). To assess the cell viability of GT, B16F10 cells were treated with various concentrations of GT (0.31–20 μg/mL) for 72 h. GT had no effect on cell viability at concentrations <2.5 μg/mL (Figure 1b). However, treatment with GT concentrations above 5 μg/mL significantly reduced the viability of the B16F10 cells (Figure 1b). Therefore, the B16F10 cells were treated with GT at concentrations of 1.25 and 2.5 μg/mL in subsequent experiments.

In the anti-melanogenesis assay, treatment with GT at concentrations of 1.25 and 2.5 μg/mL significantly reduced the melanin content of the B16F10 cells (Figure 1c).

### 2.2. Ganodermanontriol Inhibited Tyrosinase and MITF Protein Expression of B16F10 Cells

**Figure 2:**
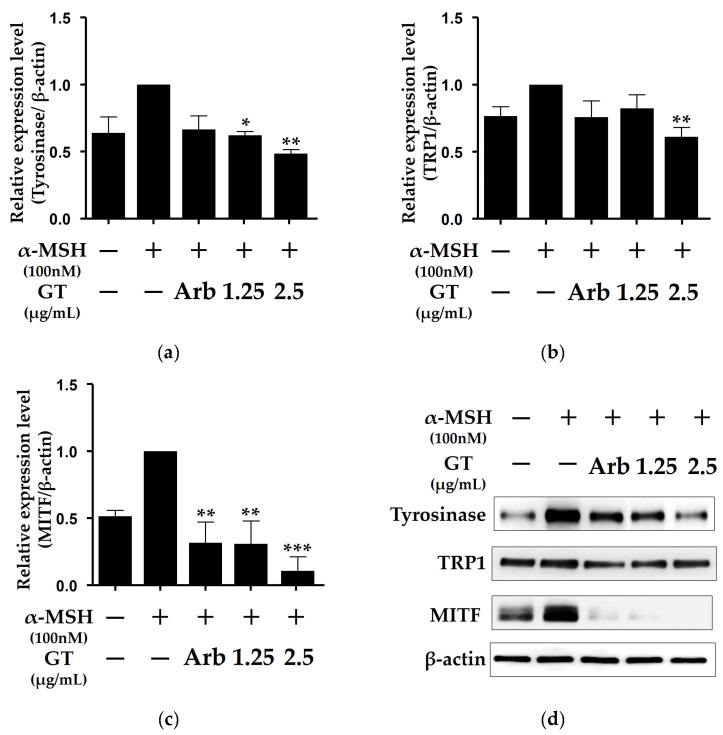
Effects of ganodermanontriol (GT) on expression of cellular tyrosinase and microphthalmia-associated transcription factor (MITF) proteins in B16F10 cells. Relative protein expression levels of tyrosinase (**a**), tyrosinase-related protein 1 (TRP-1) (**b**), and MITF protein (**c**). Relative protein levels were quantified using Western blot analysis (**d**). The data were analyzed using one-way ANOVA followed by Tukey’s test. * *p* < 0.05, ** *p* < 0.01, and *** *p* < 0.001 vs. B16F10 cells without sample treatment. Arb, arbutin (0.5 mM).

GT significantly inhibited melanin synthesis in the B16F10 cells (Figure 1c). Therefore, we evaluated the effects of GT on the expression of melanogenesis-related proteins, including tyrosinase and MITF, which play important roles in melanin synthesis in B16F10 cells [10]. Western blot analysis showed that GT at a concentration of 2.5 μg/mL significantly inhibited the expression of tyrosinase, TRP1, and MITF proteins in the B16F10 cells (Figure 2). Interestingly, GT treatment significantly reduced MITF protein expression compared with arbutin (0.5 mM) treatment (Figure 2c). These results indicated that the melanogenesis inhibitory activity of GT was associated with the inhibition of tyrosinase, TRP1, and MITF protein expression.

### 2.3. Ganodermanontriol Inhibited cAMP Response Element-Binding Protein (CREB) Phosphorylation in B16F10 Cells

**Figure 3:**
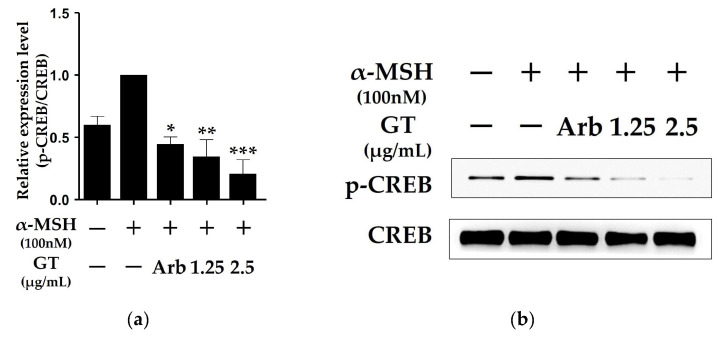
Effects of ganodermanontriol (GT) on phosphorylation of cyclic adenosine monophosphate (cAMP) response element-binding protein (CREB) in B16F10 cells (**a**). Relative phosphorylation levels were quantified using Western blot analysis (**b**). The data were analyzed using one-way ANOVA followed by Tukey’s test. * *p* < 0.05, ** *p* < 0.01, and *** *p* < 0.001 vs. B16F10 cells without sample treatment. Arb, arbutin (0.5 mM).

In the cAMP-dependent signaling pathway in B16F10 cells, CREB phosphorylation activates MITF transcription [20,21]. Additionally, the inhibition of the cAMP-dependent signaling pathway causes MITF degradation in α-MSH-stimulated B16F10 cells [22]. The phosphorylation of CREB was significantly inhibited by GT treatment in the B16F10 cells (Figure 3). In addition, the GT treatment significantly reduced the phosphorylation of CREB compared to arbutin (0.5 mM) treatment, as shown by the inhibitory effect on MITF protein expression (Figure 2c). These results suggested that the inhibition of MITF expression by GT treatment was due to the inhibition of CREB phosphorylation.

### 2.4. Effects of Ganodermanontriol on the Phosphorylation of p38, c-Jun N-Terminal Kinase (JNK), and Extracellular Signal-Regulated Kinase (ERK) Proteins in B16F10 Cells

**Figure 4:**
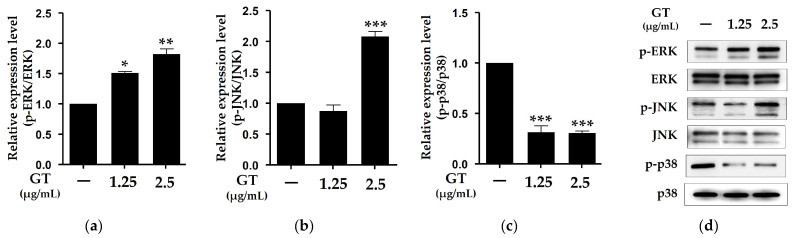
Effects of ganodermanontriol (GT) on phosphorylation of extracellular signal-regulated kinases (ERKs) (**a**), c-Jun N-terminal kinases (JNKs) (**b**), and p38 (**c**) proteins in B16F10 cells. Relative phosphorylation levels were quantified using Western blot analysis (**d**). The data were analyzed using one-way ANOVA followed by Tukey’s test. * *p* < 0.05, ** *p* < 0.01, and *** *p* < 0.001 vs. B16F10 cells without sample treatment. Arb, arbutin (0.5 mM).

Mitogen-activated protein kinase (MAPK) family proteins, including ERK, p38, and JNK, are important signaling molecules in melanogenesis [21]. Previous studies have reported that p38 phosphorylation induces MITF expression, while ERK and JNK phosphorylation inhibit melanin synthesis [21,22,23]. As shown in Figure 4, the phosphorylation of both ERK and JNK was significantly increased by the GT treatment at a concentration of 2.5 μg/mL. In contrast, p38 phosphorylation in the B16F10 cells was significantly reduced by the GT treatment at both concentrations. These results indicate that the inhibition of melanogenesis in the B16F10 cells by GT was also associated with the phosphorylation of MAPK signaling molecules, including ERK, p38, and JNK.

### 2.5. Ganodermanontriol in G. lucidum

**Figure 5:**
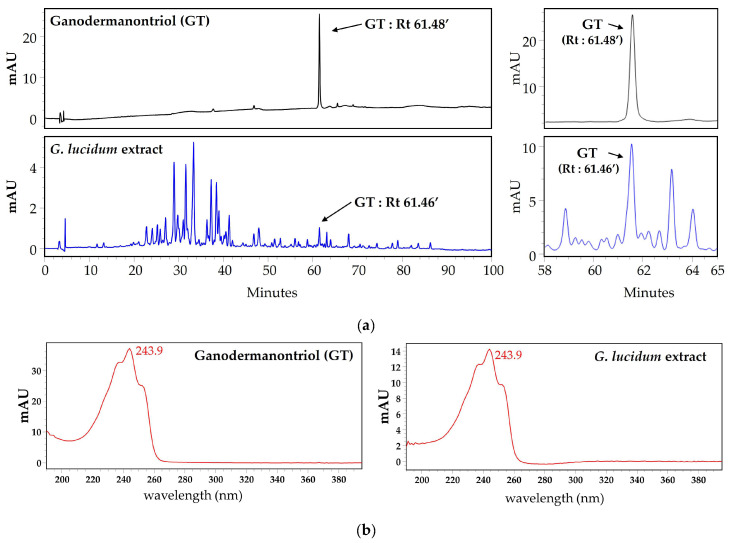
HPLC profiles (**a**) and HPLC-PDA chromatograms (**b**) of ganodermanontriol (GT) and *G. lucidum* extract. Rt, retention time. Arrows indicate GT-specific peaks.

**Figure 6:**
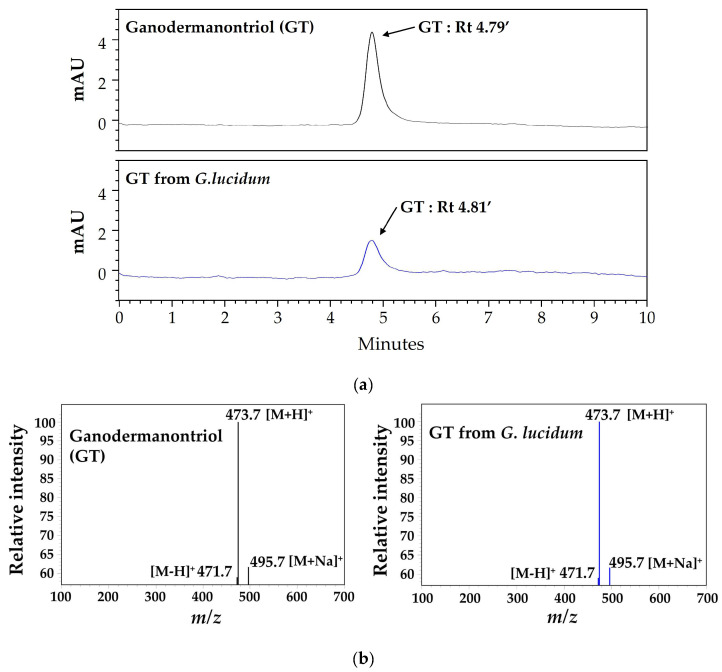
HPLC chromatograms (**a**) and ESI-MS spectra (**b**) of ganodermanontriol (GT) and GT from *G. lucidum*. Rt, retention time. Arrows indicate GT-specific peaks.

Although GT is known to be a lanostanoid triterpene derived from *G. lucidum*, high-performance liquid chromatography and analysis (HPLC) and liquid chromatography–electrospray ionization–mass spectrometry (LC-ESI-MS) analysis were performed to validate the presence of GT in this mushroom [15,16,17,18]. A single distinct peak corresponding to GT in the *G. lucidum* extract was observed in the HPLC chromatogram at a retention time of 61.46 min (Figure 5a). Figure 5b shows the chromatogram patterns of GT and the *G. lucidum* extract at 243.9 nm in the HPLC-PDA analysis. To evaluate whether this peak was a single compound for GT, single-compound isolation and LC-ESI-MS analysis were further performed. As shown in Figure 6, the LC-ESI-MS analysis revealed that the single compound corresponding to the single peak in HPLC showed fragment ions at the same *m*/*z* value 473.7 (M + H)^+^, *m/z* value 471.7 (M − H)^+^, and *m*/*z* value 495.7 (M + Na)^+^ as GT. These results demonstrate that *G. lucidum* produces a variety of compounds, including GT, as previously reported [15,16,17,18]. These results also suggest that further studies of compounds other than GT may lead to the discovery of additional substances with novel functions.

## 3. Discussion

Melanin is the main pigment that plays an important role in protecting human skin from ultraviolet (UV) radiation and solar radiation [1,24]. In this study, we demonstrated the melanogenesis inhibitory activity of GT in B16F10 cells. As shown in Figure 1c, treatment with GT significantly reduced the melanin content of the B16F10 cells, by 1.25 and 2.5 μg/mL. The intracellular melanin synthesis induced by external stimuli is influenced by various molecular factors, including tyrosinases, MITF, and MAPK kinase signaling molecules [19]. Therefore, we investigated the effects of GT on the various molecules involved in melanogenesis in B16F10 cells.

Tyrosinase is a key enzyme in melanin biosynthesis that catalyzes the rate-limiting reaction in the melanogenic pathway [1]. TRP-1 and TRP-2 determine the shape and structure of the melanosomes in melanocytes [25]. Therefore, these are important enzymes that regulate melanin biosynthesis. In the present study, GT treatment was found to significantly inhibit the expression of tyrosinase and TRP-1 protein. Because the expression of tyrosinase is regulated by the intracellular transcription factor MITF, the inhibition of this transcription factor ultimately reduces the expression of tyrosinase [10]. The MITF protein expression was also significantly decreased by GT treatment in α-MSH-stimulated B16F10 cells (Figure 2c). In addition, GT treatment at a concentration of 2.5 µg/mL inhibited MITF expression more effectively than arbutin treatment at a concentration of 0.5 mM. These results suggest that the inhibition of MITF expression by GT ultimately leads to the inhibition of tyrosinase expression in B16F10 cells, consistent with previous studies on the relationship between MITF and tyrosinase expression [10,19].

MITF expression is associated with the cAMP-dependent melanogenic pathway induced by α-MSH in melanoma cells [26]. α-MSH binds to the melanocortin-1 receptor (MC1R), leading to increased intracellular cAMP levels, which leads to the phosphorylation of CREB and subsequently increased MITF expression in melanocytes [26,27]. Additionally, inhibition of the cAMP-dependent signaling pathway degrades MITF in α-MSH-stimulated melanoma cells [21]. Figure 3 shows that CREB phosphorylation was induced in the B16F10 cells stimulated with α-MSH but was significantly inhibited by GT treatment. In addition, the 2.5 µg/mL GT treatment inhibited CREB phosphorylation more effectively than the arbutin treatment, similar to the suppression of MITF expression (Figure 2c and Figure 3). These results suggested that the inhibition of tyrosinase and MITF expression by GT was due to the inhibition of CREB phosphorylation, which, in turn, regulated their expression.

The phosphorylation of CREB inhibits melanin production in B16F10 cells and affects MAPK signaling [19]. MITF expression is induced by p38 phosphorylation, whereas melanin synthesis is inhibited by ERK and JNK [21]. MAPK signaling molecules, including ERK, p38, and c-Jun N-terminal kinase (JNK), play important roles in melanogenesis [21]. Therefore, we evaluated whether GT affected MAPK signaling molecules and CREB in the B16F10 cells. As expected, the phosphorylation of ERK and JNK proteins was significantly increased by GT treatment in the α-MSH-stimulated B16F10 cells, whereas p38 phosphorylation was significantly decreased (Figure 4). Therefore, this study demonstrated that GT from *G. lucidum* significantly reduces melanin by affecting key factors that play an important role in melanin biosynthesis in B16F10 cells.

In recent decades, various bioactive components, including triterpenes, polysaccharides, and peptidoglycan, have been reported in *Ganoderma* species [28,29,30,31,32]. Among these, approximately 500 triterpenoids have been identified, and diverse biological activities have been reported [33]. We have previously reported the anti-melanogenic effects of ganodermanonediol (GN) from *G. lucidum* [19]. In our previous study, GN showed an anti-melanogenic activity of 93.5% at a concentration of 2.5 µg/mL, but in this study, GT showed an inhibitory activity of 49.6% at the same concentration. Despite these differences in efficacy, GT has a very similar molecular structure to GN, with one additional hydroxy group at C-7. This suggests that it can be used as information to understand the relationship between the structure and activity of various substances in *G. lucidum*.

GT has been reported to be a triterpenoid uniquely found in *G. lucidum* and was also identified in this study by HPLC and LC-ESI-MS analyses [15,16,17,18] (Figure 5 and Figure 6). Previous studies have reported that GT from *G. lucidum* inhibits the expression of CDC20, which controls mitotic chromosome segregation during the proliferation of human breast cancer cells [15]. Additionally, GT has been reported to have an inhibitory effect on the HIV-1 virus [18]. However, to our knowledge, this is the first study to demonstrate that GT inhibits the expression of melanogenesis-related proteins and affects the phosphorylation of CREB and MAPK signaling molecules in B16F10 cells, resulting in anti-melanogenic effects.

## 4. Materials and Methods

### 4.1. Reagents

Ganodermanontriol (GT) was purchased from ChemFaces (Seoul, Republic of Korea). Dulbecco’s modified Eagle’s medium (DMEM), fetal bovine serum (FBS), and antibiotics (penicillin and streptomycin) were purchased from Gibco BRL (Grand Island, NY, USA). The antibodies used in this study were purchased from Cell Signaling Technology (Danvers, MA, USA), while 3-(4,5-Dimethylthiazol-2-yl)-2,5-diphenyltetrazolium bromide (MTT), α-MSH, and all the other chemicals were purchased from Sigma-Aldrich (St. Louis, MO, USA). The B16F10 murine melanoma cells (CRL-6475) were obtained from the American Type Culture Collection (ATCC, Manassas, VA, USA).

### 4.2. Ganoderma lucidum and Ganodermanontriol Isolation

**Figure 7:**
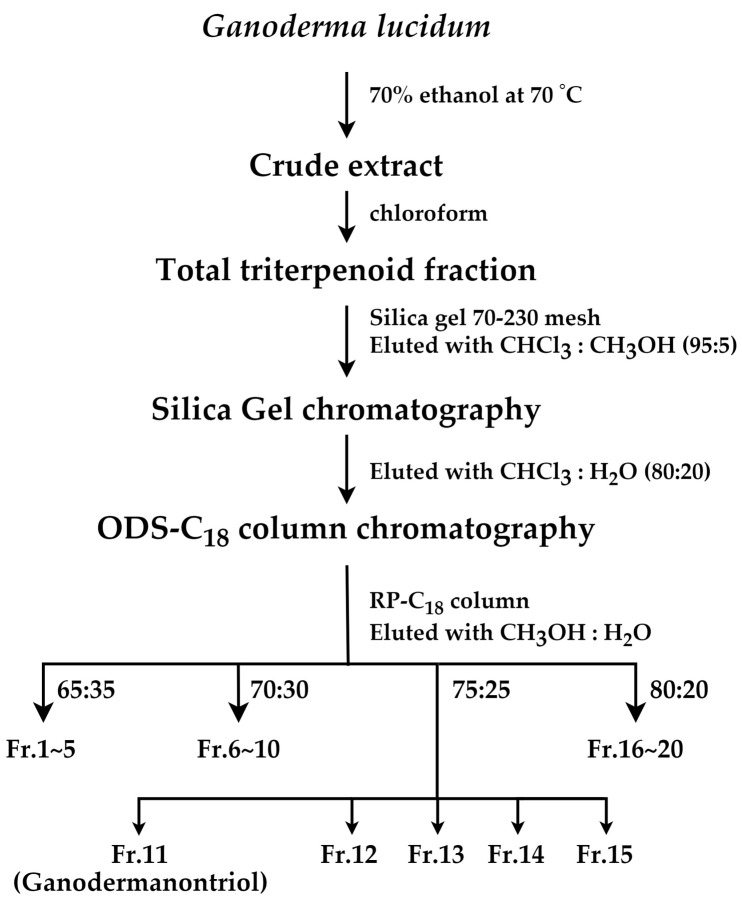
Isolation procedure of ganodermanontriol from *Ganoderma lucidum*. The purification was started with the fruit body of *G. lucidum*. Each step is indicated by one arrow.

*G. lucidum* ASI 7071 was obtained from the Mushroom Research Division of the National Institute of Horticultural and Herbal Science (Rural Development Administration, Eumseong, Republic of Korea). The isolation of GT from *G. lucidum* was performed using a previously described method [16]. Briefly, *G. lucidum* (4 kg) was extracted with 70% ethanol (30 L) at 70 °C, and the extract (90 g) was treated with chloroform (2 L) to obtain a triterpenoid fraction (19 g). The triterpenoid fraction was applied to a silica gel (70–230 mesh, Merck Millipore, Darmstadt, Germany) column eluted successively with chloroform:methanol (95:5). The fraction was subjected to octadecylsilane (ODS)-C_18_ column (YMC, Kyoto, Japan) chromatography eluted with methanol:water (80:20) and further separated using reverse-phase (RP) C_18_ (Merck Millipore, Darmstadt, Germany) column chromatography (methanol:water, 75:25) to obtain GT (Figure 7).

### 4.3. Cell Culture and Viability Assay

The B16F10 cells were cultured in DMEM supplemented with 10% fetal bovine serum (FBS) and 1% penicillin–streptomycin. The cells were incubated and maintained in a 5% CO_2_ incubator at 37 °C. The viability of the B16F10 cells was determined using an MTT assay, as described previously [19]. Briefly, cells treated with GT (0.31–20 μg/mL in dimethylsulfoxide; DMSO) were incubated with MTT solution (50 mg/mL) for 3 h, and the optical density of DMSO-dissolved formazan crystals was then measured at 570 nm using a microplate reader (BioTek Epoch2, Agilent, Santa Clara, CA, USA).

### 4.4. Melanin Content Assay of the B16F10 Cells

The melanin content was determined using a previously described method [19]. Briefly, B16F10 cells treated with GT (1.25 and 2.5 μg/mL) for 72 h were washed with phosphate-buffered saline (PBS) and then incubated in a 1 M sodium hydroxide (NaOH) solution at 60 °C for 1 h. The relative melanin content was measured at 405 nm using a microplate reader (BioTek Epoch2, Agilent, Santa Clara, CA, USA).

### 4.5. Western Blot Assay

B16F10 cells treated with GT were lysed using radioimmunoprecipitation assay (RIPA) buffer (Thermo Scientific, Seoul, Republic of Korea) and centrifuged at 10,000× *g* for 10 min, and the protein concentration was measured using Bradford assay reagent (Bio-Rad Korea, Seoul, Republic of Korea). Western blot analysis of the total protein was performed as previously described [19]. Primary antibodies against tyrosinase, tyrosinase-related protein-1 (TRP-1), microphthalmia-associated transcription factor (MITF), p38, p-p38, extracellular signal-regulated kinase (ERK), p-ERK, c-Jun N-terminal kinase (JNK), p-JNK, cAMP response element-binding protein (CREB), and p-CREB were diluted at a ratio of 1:1000. The signals were quantified using a C-DiGit Blot Scanner (LI-COR Korea, Gyeonggi-do, Republic of Korea).

### 4.6. High-Performance Liquid Chromatography Analysis and Liquid Chromatography–Electrospray Ionization–Mass Spectrometry

The HPLC analysis was performed using a Waters 2690 Alliance HPLC system (Waters Corporation, Milford, MA, USA) with a C_18_ analytical column (Phenomenex Luca C_18_ 100 Å, 250 × 4.6 mm, 5 μm particle size, Phenomenex Co., Torrance, CA, USA) at 254 nm. Chromatographic separation was performed using a gradient program with two solvents (Table 1). The LC-ESI-MS analysis was performed using an Agilent Infinity Lab LC/MSD system (Agilent Technologies, Santa Clara, CA, USA) with a C_18_ analytical column (mobile phase: isocratic elution with methanol:water, 75:25) at 254 nm. The analytical conditions were as follows: ion source: API-ES; scan range: *m****/****z* 100–700; positive polarity; fragmentor: 250 V; Nebulizer gas: 60 psi; gas temperature: 300 °C. The data acquisition and analysis were carried out using OpenLab CDS ChemStation Edition (Agilent Technologies, Santa Clara, CA, USA).

## Figures and Tables

**Table 1:** Parameters of gradient elution for HPLC analysis.

Time (min)	Flow Rate	Solvent A	Solvent B
	(mL/min)	Water Containing 0.2% Acetic Aid	Acetonitrile
0	0.8	80	20
20	0.8	65	35
30	0.8	60	40
40	0.8	50	50
50	0.8	40	60
55	0.8	30	70
60	0.8	25	75
70	0.8	15	85
80	0.8	5	95
90	0.8	0	100
100	0.8	0	100

## Data Availability

The original contributions presented in the study are included in the article, further inquiries can be directed to the corresponding author.
